# Effects of exposure to immersive computer-generated virtual nature and control environments on affect and cognition

**DOI:** 10.1038/s41598-022-26750-6

**Published:** 2023-01-05

**Authors:** Fariba Mostajeran, Marvin Fischer, Frank Steinicke, Simone Kühn

**Affiliations:** 1 Department of Informatics, Human-Computer Interaction Group, Universität Hamburg, 22527 Hamburg, Germany; 2 Clinic and Polyclinic for Psychiatry and Psychotherapy, Neural Plasticity Group, University Medical Center Hamburg-Eppendorf, 20246 Hamburg, Germany; 3 Max Planck Institute for Human Development, Lise Meitner Group for Environmental Neuroscience, 14159 Berlin, Germany

**Keywords:** Computer science, Psychology

## Abstract

Previous research has shown that exposure to immersive virtual nature environments is able to induce positive affective and physiological effects. However, research on the effects on cognitive performance is scarce. Additionally, the effects of virtual nature exposure compared to a virtual control environment with a comparable amount of virtual objects have not been examined so far. Therefore, we conducted an experiment with 27 participants to study the psychological effects of such exposure. The virtual nature consisted of a 3D model of a typical forest environment, whereas the control environment was an abstract replication of the virtual forest environment. In both environments, a virtual wooden cart was used to transport the users from the start to the end of the virtual road. The typical background noise of moving such a cart was integrated into both environments as well. In addition, the virtual nature environment included typical forest sounds in the background, whereas the control condition did not have such background sounds. Both environments were compared with regard to their effects on cognitive performance (using trail making tests (TMTA, TMTB, and TMTB-A) as well as digit span forward and digit span backward tests), perceived restorativeness, mood, stress, sense of presence, and simulator sickness. The results showed that in comparison to the control environment, exposure to the virtual nature resulted in significantly higher cognitive performance, higher perceived restorativeness, higher positive affect, higher sense of presence, lower perceived stress, and lower simulator sickness.

## Introduction

Extensive empirical research has demonstrated evidence for positive effects of nature on human’s mental and physical health ^1–5^. These benefits span from positive effects on mental processes^6^ to physical functions^7^, social interactions^8^, and even tangible benefits associated with wealth (e.g. a public park can influence the sale price of nearby homes)^9^. Positive effects on mental processes have been shown, for instance, through reductions of aggression^10^ and anxiety^11^ as well as increased self-esteem^12^, improved mood^13,14^, psychological well-being^6^ and behavior^15^. These effects have also been objectively measured in physiological reactions such as reduced blood pressure^16^ and the stress-related cortisol hormone^17^. Moreover, as potential long-term effects of nature, occurrence of illnesses has been shown to be reduced as a result of interaction with nature^7,18^.

Another outcome of interaction with nature is its positive effect on cognitive ability and functions^19,20^. Cognitive functions refer to several mental abilities such as learning, problem-solving, memory, and attention^21^. Directed or voluntary attention describes the ability to focus on a task that requires effort^19^. However, this cognitive resource or ability is not infinite and may become fatigued^22^. For instance, one can experience attention fatigue while focusing on a task with little or no intrinsically motivational draw when simultaneously having to suppress more interesting input^23,24^. A suggested remedy is to take a break from the task and spend time in natural environments^22^. Attention restoration theory (ART)^6,23^ is one theory that provides an explanation for this effect. It proposes that nature, which is full of intriguing stimuli, grabs attention in a bottom-up fashion allowing for top-down directed-attention abilities to be restored. Several studies have validated this theory by showing improvements in the performance of cognitive tasks, in particular, in tests assessing executive functioning such as Trail Making Test and Digit Span Tests, after exposure to nature^22,25^.

Similar positive effects have been observed for illustrations or simulations of nature as well^26,27^. These span from simple photographs to fully immersive virtual reality (VR) environments. For instance, Berman et al.^20^ found that cognitive performance was improved after viewing photographs of natural scenes. Simulations of natural environments are in particular beneficial for situations when access to real nature is limited. Older adults in nursing homes, hospital patients, and prison inmates are exemplary individuals with limited access to nature. However, COVID-19 pandemic forced more individuals globally, especially in urbanized environments, into prolonged lockdowns and likewise restricted their access to natural environments. Recent studies have shown that these reduced interaction with nature, especially green spaces such as parks, were linked to fears about the virus and were associated with higher levels of emotional distress^28,29^.

The advantage of immersive VR compared to other types of natural environment simulations is its ability to provide a sense of presence (i.e., an illusion of being physically present) in the virtual nature^30,31^. Typically, immersive VR systems exploit a head-mounted display (HMD), which displays a virtual environment (VE) such as a virtual nature setting, while blocking the users’ field of view to the real, physical world. The combination of multiple sensory channels such as the human’s visual and auditory senses, which can be stimulated by the VR system enables users to perceive, feel, and interact in the virtual nature similar as in real nature environments^32^.

This perceived sense of presence in the VE is typically positively correlated with the level of immersion, which describes the objective properties of a VR technology. This means that a VR system, which is capable of delivering an inclusive, extensive, surrounding and vivid illusion of reality, will likely produce a higher psychological state of feeling present in the VE^31^. Accordingly, photo-realistic computer-generated VEs are objectively more immersive and may lead to higher sense of presence compared to other types of visual contents displayed via a VR system such as $360^{\circ }$ photos and videos. The reason is that they provide only limited capability for intuitive exploration as only head orientations supported head-gaze rendering, whereas parallax effects due to positional changes are not supported^32^.

Previous studies on nature simulation in VR have employed various visual stimuli with levels of immersion including panoramic photos^33^, $360^{\circ }$ photos^34–36^ and videos^37,38^, and computer-generated VEs^27^. The environments have been also diverse and varying from indoor green office designs^39^, green roadsides^40^, urban green spaces and parks^35^ to national forests^41^, beaches^42^, and underwater worlds^43^.

Comparisons between immersive VR and real nature exposures have revealed that VR can induce similar positive effects as real nature^44–48^. For example, Browning et al.^44^ compared exposure to a real forest and a $360^{\circ }$ video of the same environment and observed that both were more restorative in comparison to a physical indoor environment without nature. In addition, the sense of presence was not reported to be significantly different between $360^{\circ }$ videos and real nature (a lake) environment^49^. In fact, participants (N = 100) of a study conducted by Mattila et al.^50^ viewed computer-generated nature (a forest) in VR as restorative as the real natural environments, and yet, more fascinating and coherent.

Positive physiological and affective outcomes of exposure to immersive VR nature have been studied in previous research^51^. For instance, Wang et al.^52^ demonstrated that exposure to $360^{\circ }$ videos of different types of forest environments can improve mood and relieve stress. Recovery from stress as a result of exposure to virtual nature has been also demonstrated by Annerstedt et al.^53^, who showed that recovery is enhanced when the environment is presented together with natural sounds. Similar effects were observed in other studies which demonstrate that visual stimuli of virtual nature are more effective for stress reduction when accompanied by auditory stimuli (e.g., bird songs)^36,54^.

Physiological arousal and negative affect have also been shown to be reduced as a result of exposure to immersive virtual nature^55^. Significant reduction of anxiety^56,57^, negative emotions such as fatigue and depression^41^ and pain relief^58^ have been reported as well. In addition, exposure to virtual nature has demonstrated significant increase of positive affect^59^ and has been used as a mood-induction procedure for inducing relaxation^57^. For these reasons, it has been suggested that exposure to virtual nature could be used by the general population for relaxation purposes especially during pandemic when stress seems to have increased globally^60^.

However, research on the effects of exposure to immersive virtual nature on cognition is relatively scarce^51^. There are only a few studies, which have included cognitive measures as an outcome variable of exposure to virtual nature. In one of them, presented by Chung et al.^61^, brain activity was recorded using electroencephalography (with the goal to capture involuntary attention restoration) while participants performed an auditory oddball task. Their results showed that in comparison to $360^{\circ }$ videos of fireworks, exposure to $360^{\circ }$ videos of natural environments (seaside, grassland, and hilly scenes) improved cognitive functioning and restored involuntary attention. In another study, Valtchanov et al.^62^ administered mental-arithmetic quizzes (i.e., five multiplication and five division questions), but could not observe significant differences in math performance before and after exposure to an immersive computer-generated virtual forest environment. Nevertheless, increased positive affect and decreased stress could be observed. A possible reason for the lack of cognitive improvement could be the level of difficulty of the chosen tasks, as they might have been too easy to detect any subtle changes in performance.

In a series of studies^39,48,63^, Yin et al. examined the effects of computer-generated green office designs on psychological responses, including cognition. In one of their studies, they observed a 14% improvement of short-term memory due to exposure to a green office in VR^48^, whilst in another study, negative impact was observed as participants needed longer reaction times for performing a cognitive test (i.e., Stroop test)^63^. Yu et al.^64^ also reported no significant changes in participants’ attentional capacities (measured by Sustained Attention to Response Test) before and after exposure to $360^{\circ }$ videos of various forest environments. Although the middle-aged and older participants of their studies perceived the virtual nature to be more relaxing and reported higher perceived restorativeness and lower fatigue and depression after viewing nature scenes.

Finally, Mostajeran et al.^37^ recently showed improvements in cognitive performance after exposure to $360^{\circ }$ videos of a forest environment. As a cognitive test users were asked to serially subtract the number 13 from a given starting number (e.g., 1022). It could be shown that the maximum number of correct answers (number series) in the conditions exposing to the virtual forest environment was higher and the total number of errors was lower. Thus, the effects of immersive virtual nature exposure on cognitive functions are not clearly understood. Some studies have shown improvements in short-term memory and mental arithmetic performance while others have failed to observe any changes in attention and math performance. In addition, not all of these studies have administered classical cognitive tests to quantify the effects of exposure to real nature^22^.

Moreover, outdoor natural environments have been often presented via real-life $360^{\circ }$ videos in previous studies. Although, Nukarinen et al.^38^ have suggested that a computer-generated model of a real natural environment might be more emotionally restorative than a $360^{\circ }$ video of the same environment. Besides, $360^{\circ }$ videos of natural environments do not grant full control over the visual stimuli. Computer-generated VEs, on the other hand, open up vast opportunities to systematically model and control all elements of virtual nature such as seasonal changes, weather conditions, vegetation, and even subtle movements of leaves and grass in order to unravel the “active ingredients” of the observed effects. Additionally, comparable virtual control environments with a similar amount of virtual objects can be easily created for computer-generated virtual natures. However, such a comparison between a computer-generated virtual control and nature environment has not been undertaken so far^65^.

Therefore, we designed an experiment to examine the effects of exposure to a computer-generated nature environment in comparison to a virtual control environment. Participants’ cognitive performance after exposure to the virtual nature environment was compared to their performance after exposure to a neutral control VE. We intentionally designed a post-test-only experiment as previous studies (including one of ours^37^) have included a baseline condition and have shown improvements in the dependent variables after the virtual nature exposures compared to the baseline. The main question of this study was not the improvements of the measures compared to the baseline but rather a paired comparison of the post-test measures of the two virtual environments. Our reasoning was that multiple repetitions of the cognitive tests could be tiring for participants and this may introduce additional noise. In this study, the virtual nature consisted of a typical forest environment, whereas the control environment was an abstract shape representation of the forest environment. Both environments were compared with respect to their effects on not only cognitive performance but also perceived restorativeness, mood, stress, sense of presence, and simulator sickness. To measure cognitive performance, we employed four neuropsychological tests, which have shown empirical evidence to support ART after exposure to real nature^22,25^. We hypothesized that in comparison to the control environment, exposure to virtual nature results in **(H1)** higher cognitive performance, **(H2)** higher perceived restorativeness, **(H3)** higher positive affect (PA), **(H4)** lower negative affect (NA), **(H5)** lower stress, **(H6)** higher sense of presence and **(H7)** lower simulator sickness.

## Methods

### Participants

Prior to starting the experiment, we performed sample size calculation using G*Power^66^ with a power of .8, an effects size of .5, and an alpha error probability of .05, which resulted in 27 participants that were recruited for the experiment. We recruited participants via an email distributor among the students of the Department of Computer Science at the University of Hamburg as well as paper advertisements. A total of 27 individuals (12 women) between 21 and 59 years of age ($M=28.15, SD=8.27$) participated in the study, from whom 16 had prior experience with VR. Two participants reported color blindness and two others stated that they have attention deficit disorder (ADD) or attention deficit hyperactivity disorder (ADHD). The analysis of data was performed once with and once without the two participants who reported to have ADD or ADHD. As the pattern of findings were similar in both cases, the final report includes the data of these two participants. The study was approved by the local psychological ethics committee of the Center for Psychosocial Medicine at the University Medical Center Hamburg-Eppendorf and was carried out in accordance with relevant guidelines and regulations.

### Virtual environments

**Figure 1:**
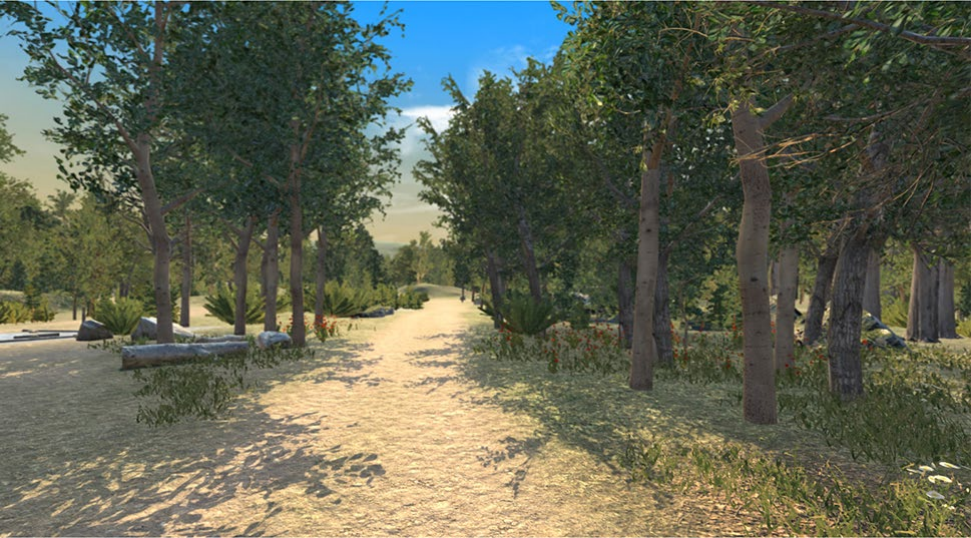
Nature environment.

In this study, we were particularly interested in understanding the effects of a natural environment in comparison to a neutral environment with a comparable amount of virtual objects in similar sizes and shapes. Therefore, two VEs were designed and implemented using the Unity game engine. The virtual nature environment included typical computer-generated visual elements of a forest such as trees and bushes (see Fig. 1) as well as background sound of a typical forest with the sound of singing birds. We made use of several 3D models and materials to build our virtual nature environment mainly from two assets from Unity Asset Store, namely “Nature Starter Kit 2” and “Meadow Environment - Dynamic Nature” We used mostly 3D models of Poplar trees which are native to most of the northern hemisphere.

**Figure 2:**
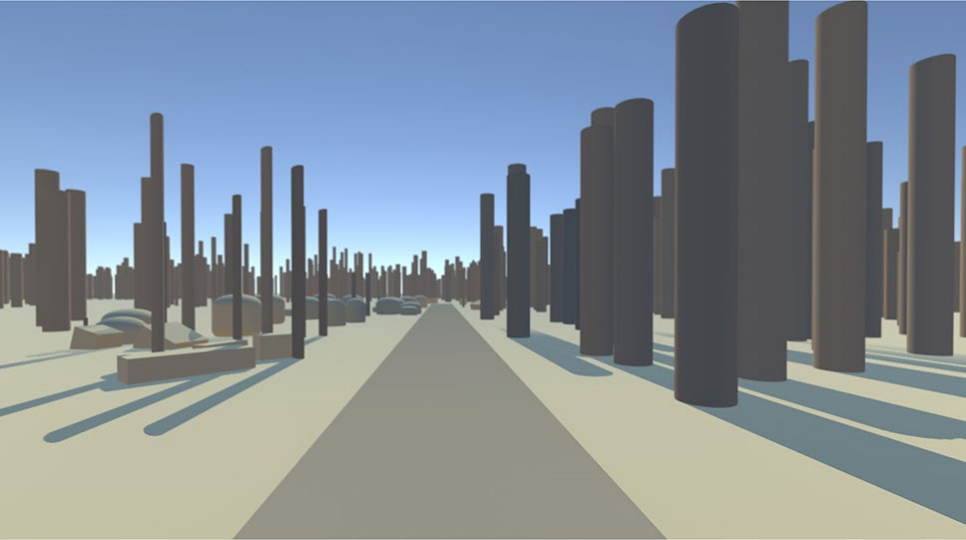
Control environment.

Our design of the control environment was inspired by the work of Llobera et al.^67^ on social behaviours towards virtual humans. In their study, virtual humans were compared with a cylinder of human size when either of these virtual representations approached participants. Therefore, our control condition (see Fig. 2) consisted of abstract objects such as cylinders, spheres and cuboids. Each element in the nature environment was replaced with an abstract object in the control environment. For example, a cylinder was placed in the control environment at each tree location in the nature environment. Rocks and bushes were also placed with spheres and cuboids in the control environment.

The choice of abstract objects was made during the design phase as the use of neutral but similarly familiar objects from urban environments such as street light poles instead of trees in an urban setting would recreate an urban setting or in a neutral setting as ours would not provide such a degree of neutrality as our current design gave us. Therefore, we intentionally prevented creating another urban environment or a mix of abstract and urban environments. Instead, we aimed at creating a completely neutral environment as much as possible. Such an environment is also not completely unfamiliar to the participants from our digital era. Abstract objects have been constantly used in computer-generated media such as games and movies.

In both environments, a virtual wooden cart was used to transport the users from the start to the end of the virtual road (10 min per condition). The typical background noise of moving such a cart was integrated into both environments as well. Therefore, in the control environment, this noise was the only sound that could be heard by the users. We considered the sound of a moving wooden cart a more neutral stimulus than any other transporter we could use for moving the user from the start to the end of the road. That is why this sound was added to both environments. To display the VR content, an HTC Vive Pro HMD with integrated headphones was employed. The resolution of this HMD for each eye is 1440 × 1600 pixels with a refresh rate of 90 Hertz.

### Measures

The following cognitive tests and questionnaires were employed in this study. As measures of reliability, Cronbach’s $\alpha$ and McDonald’s $\omega _t$ were calculated and reported for each questionnaire and for each condition (Nature and Control). The reliability of a self-reported measure is commonly interpreted as acceptable if it is greater than or equal to 7^68–70^.

*Trail Making Test (TMT)*^71,72^ Assesses an array of cognitive domains and comprise parts A and B (i.e., TMTA and TMTB). In part A, participants use a pencil to connect a series of 25 encircled numbers in numerical order (e.g., $1 \rightarrow 2 \cdots \rightarrow 25$). In part B, participants are supposed to connect 25 encircled numbers and letters in numerical and alphabetical order, occurring in turn between the numbers and letters (e.g., $1 \rightarrow A \rightarrow 2 \rightarrow B\cdots \rightarrow 13$). The primary outcome of the test is the required time in seconds to completion for TMTA and TMTB. Thus, longer times represented worse cognitive function and the typical maximum score and cutoff time is set at 300 s. Before the measurement, participants receive an exercise with eight circles in order to become familiar with the two types of test. In general, TMTA taps into cognitive domains such as visual search, attention, and psychomotor speed while TMTB additionally includes executive functions such as task switching and higher working memory load ^22,71–73^. Therefore, executive function can be measured by excluding psychomotor speed through subtracting the time to complete TMTA from TMTB (TMTB-A)^74^. The analog test according to Ralph M. Reitan was used because digital versions may not have the same validity as the analogue version^71,75^ and the analogue version has been frequently used in previous studies.

*Digit Span Memory Tests (DSM)*^76^ Measure short-term^77,78^ working memory^79^. In its visual version, a sequence of digits (0–9) appears on the computer screen. Each digit is displayed for one second. The participant has to remember the sequence of numbers and repeat them either in the presented order (i.e., digit span forward (DSF) test) or in reverse order (i.e., digit span backward (DSB) test). If the answer is correct the next sequence of numbers presented is one digit longer. If the answer is incorrect, the same length appears again. If an incorrect entry is made twice in succession, the length of the number sequence is reduced by one. In DSF, the first sequence of numbers is three digits long, while in DSB the initial length is two digits. The result is the longest sequence of numbers that the participant was able to correctly repeat before making two errors in succession. The participant completes DSF first and then proceeds with DSB.

*Perceived Restorativeness Scale (PRS)* Assesses perceived restorativeness and is relevant to query different aspects of ART. The short German version of this test^80^ has 12 items which can be rated on a 11-point Likert scales (0 = Not at all, 10 = Completely). The items form five sub-scales (BA = Being Away, COM = Compatibility, COH = Coherence, FA = Fascination, SCO = Scope) and a total score. The test showed acceptable to excellence reliability scores ($\alpha =.77-.85, \omega _t=.88-.92$).

*Positive and Negative Affect Schedule (PANAS)*^81^ Uses 20 adjectives to assess the current positive and negative affects (PA/NA). The response scale ranges from 1 (Very slightly/not at all) to 5 (Extremely). The reliability was to excellent (PA: $\alpha =.9-.92, \omega _t=.94-.95$, NA: $\alpha =.6-.79, \omega _t=.89-.93$).

*Perceived Stress Scale (PSS)*^82^ Is a self-report measure for stress with a good to excellence reliability ($\alpha =.86, \omega _t=.91$). It contains 10 items with the response scale ranging from 0 (Never) to 4 (Very often). As the original items refer to the situations during the past month in one’s life, for the purpose of this study, the items were modified to measure the momentary perceived stress.

*Igroup Presence Questionnaire (IPQ)*^83^ Was used to measure the perceived sense of presence in VR. It contains 14 items on a 7-point Likert scale ranging from 0 to 6 with different scale anchors, meaning that some items have general scale anchors (0 = Fully disagree to 6 = Fully agree) and some have more precise anchors (e.g., 0 = Not consistent and 6 = Very consistent). The questionnaire has four sub-scales: General Presence or the Sense of Being There, Spatial Presence, Involvement and Experienced Realism. The questionnaire showed a good to excellent reliability ($\alpha =.81-.89, \omega _t=.88-.92$).

*Simulator Sickness Questionnaire (SSQ)*^84^ Assesses 16 symptoms that may occur during or after VR exposure. The symptoms are rated from 0 (None) to 3 (Strong) and are subsumed into three subscales: Nausea, Oculomotor and Disorientation. The total SSQ score has a good to excellent reliability score ($\alpha =.84-.88, \omega _t=.9-.93$).

### Procedure

The experiment followed a within-subject design. At the beginning, the experimenter introduced the procedure of the experiment to each participant. The participants were informed that they will experience two virtual environments via a VR HMD and after each exposure, there will be some tests and questions they have to answer. The order of the two environments was not revealed to them. After signing the informed consent, participants sat on a firm chair in a laboratory room and wore the HMD. During the setup time, the HMD showed a black, empty space with grid lines. Furthermore, it was emphasized that in case of physical discomfort the VR session could be interrupted at any time. The participants were instructed to sit back and freely observe their surroundings as they are moved through the virtual environment until the End sign appears on the scene. When ready to start, each participant started with the first VR environment which lasted for 10 min. After that participants took off the HMD and took the cognitive tests (i.e., TMTA, TMTB, DSF, and DSB) and filled out the questionnaires (SSQ, IPQ, PRS, PANAS, and PSS). Thereafter, exposure to the second environment was started. Both conditions were completed in a randomized order (between-subject). After the second condition, participants filled out the demographic questionnaire and were debriefed. In addition, they were given the opportunity to ask any questions they had about the study which were answered and clarified. In total, the experiment lasted for about 90 min.

### Data analysis

**Figure 3:**
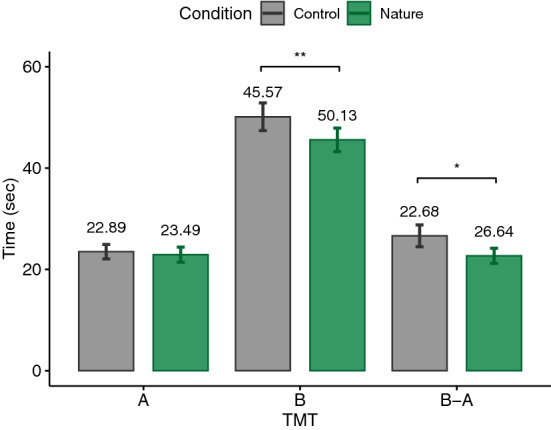
Trail making tests (TMT).

**Figure 4:**
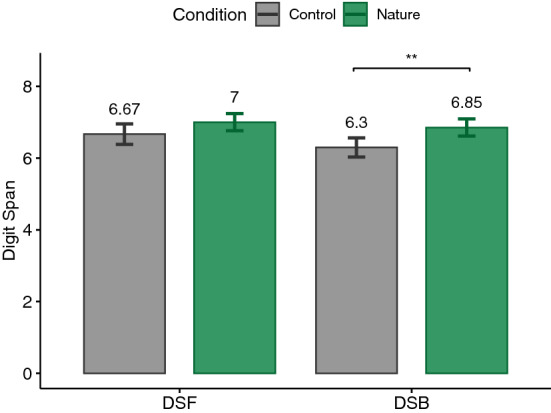
Digit span forward and backward (DSF/DSB) memory tests.

**Figure 5:**
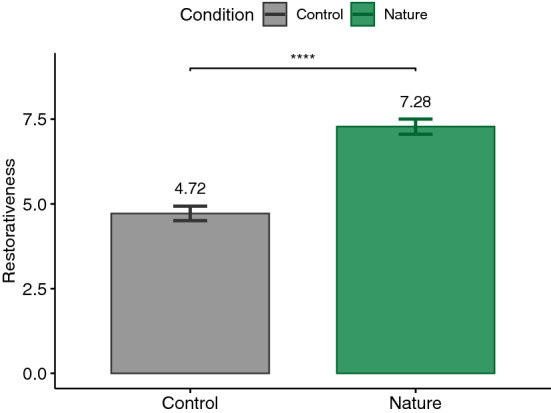
Perceived restorativeness scale (PRS) total score.

**Figure 6:**
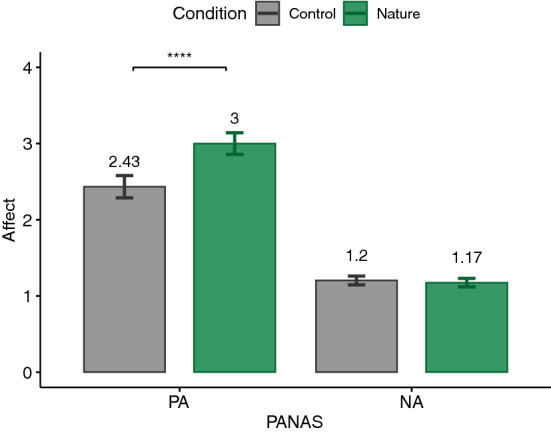
Positive and negative affect schedule (PANAS), PA: positive affect, NA: negative affect.

**Figure 7:**
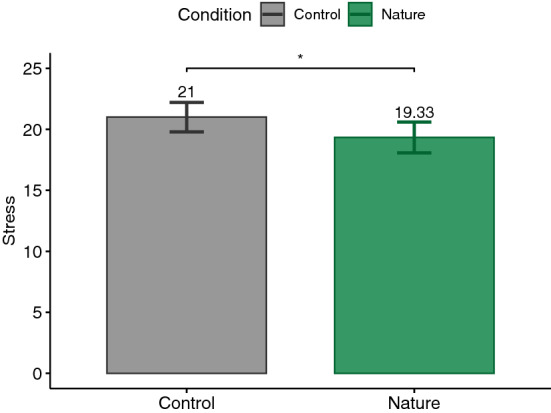
Perceived stress scale (PSS).

**Figure 8:**
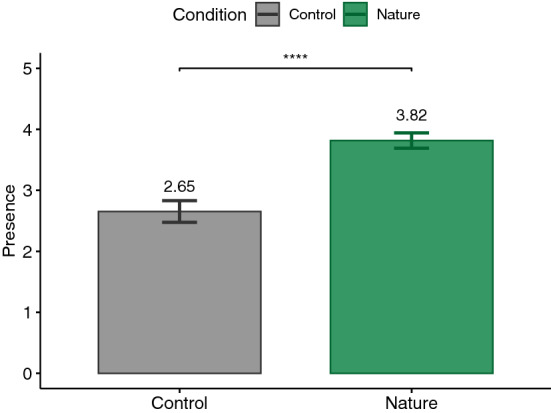
Igroup presence questionnaire (IPQ).

**Figure 9:**
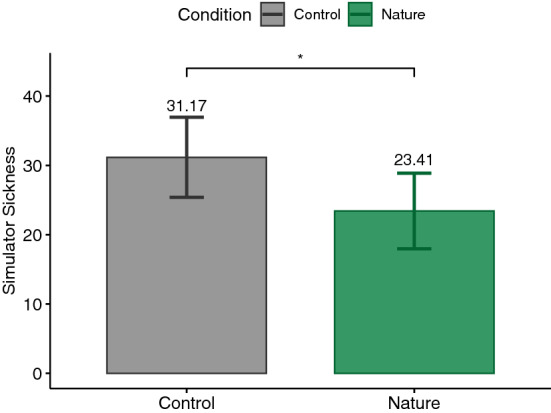
Simulator sickness questionnaire (SSQ) total score.

According to the Shapiro–Wilk test, some of our data were normally distributed and some were not. Therefore, we decided to report the analysis based on parametric tests in order to not switch between statistical tests. For this reason, for each dependent variable and according to our hypothesis, a paired one-tailed t-test was calculated. The significance level was set at .05. Our analyses followed a preregistered protocol (https://aspredicted.org/PNW_NYR). Nevertheless, performing paired two-tailed tests on the measures did not change the significance of the results, except for PSS which yielded a *p*-value of .057, suggesting a trend toward our hypothesis (see supplementary Table). Additionally, Cohen’s *d* was reported as the effect size for t-test which is commonly interpreted as small ($|d| = .2$), medium ($|d| = .5$), and large ($|d| = .8$) effects^85^. The main results are plotted in Figs. 3, 4, 5, 6, 7, 8 and 9 where asterisks represent *p*-values (* $p < .05$, ** $p < .01$, *** $p < .001$, **** $p < .0001$).

## Results

### TMT

In addition to the completion time for TMTA and TMTB, a difference score for the time needed to complete TMTB subtracted by the time needed to complete TMTA was calculated to obtain a purer estimate of executive functioning. For all three t-tests, it was tested whether the values were less after exposure to the virtual nature condition. The results suggest that participants were significantly faster in the TMTB ($t(26)=-2.34, p=.01, |d|=.45$) as well as TMTB-A ($t(26)=-2.18, p=.02, |d|=.42$) after exposure to the virtual nature environment (TMTB: $M=45.57, \textit{SD}=12.11$, TMTB-A: $M=22.68, \textit{SD}=7.81$) compared to the control environment (TMTB: $M=50.13, \textit{SD}=14.15$, TMTB-A: $M=26.64, \textit{SD}=11.19$). Thus, H1 could be confirmed by the results of TMTB and TMTB-A (see Fig. 3). No significant differences could be observed between TMTA values ($t(26)=-.5, p=.31, |d|=.095$) after nature ($M=22.89, \textit{SD}=7.77$) and control ($M=23.49, \textit{SD}=7.42$) environments.

### DSM

A t-test was performed for each of DSF and DSB to check whether the digit span was longer after exposure to the nature condition. No significant differences could be observed between performance in DSF ($t(26)=1.06, p=.15, |d|=.2$) after exposure to nature ($M=7, \textit{SD}=1.24$) and control ($M=6.67, \textit{SD}=1.49$) environments. A significant longer digit span ($t(26)=2.96, p=.003, |d|=.57$) after exposure to the virtual nature environment ($M=6.85, \textit{SD}=1.23$) compared to the control environment ($M=6.3, \textit{SD}=1.38$) could be observed for DSB (see Fig. 4) which supports H1.

### PRS

For the total PRS score, a t-test was used to test the H2 hypothesis of higher values after exposure to the nature condition ($M=7.28, \textit{SD}=1.16$) compared to the control condition ($M=4.72, \textit{SD}=1.11$). The result ($t(26)=10.13,p<.0001,|d|=1.95$) supports H2 (see Fig. 5). We repeated the analysis for each sub-scale of the PRS and found significantly higher scores for Being Away ($t(26)=10.27, p<.0001, |d|=1.98$), Compatibility ($t(26)=10.26, p<.0001, |d|=2.04$), Coherence ($t(26)=2.23, p=.02, |d|=.43$), and Fascination ($t(26)=11.48, p<.0001, |d|=2.21$) after exposure to virtual nature environment (Being Away: $M=7.94, \textit{SD}=1.19$, Compatibility: $M=7.19, \textit{SD}=1.84$, Coherence: $M=6.26, \textit{SD}=1.19$, Fascination: $M=7.91, \textit{SD}=1.58$) compared to the control environment (Being Away: $M=4.87, \textit{SD}=1.96$, Compatibility: $M=2.81, \textit{SD}=1.55$, Coherence: $M=5.53, \textit{SD}=1.72$, Fascination: $M=3.27, \textit{SD}=1.83$). No significant differences ($t(26)=0, p=.5, |d|=0$) could be observed for the Scope sub-scale (Nature: $M=7.11, \textit{SD}=2.26$, Control: $M=7.11, \textit{SD}=1.72$).

### PANAS

It was tested whether PA was higher and NA was lower after exposure to the nature environment. As it can be seen in Fig. 6, a significantly higher PA ($t(26)=5.16, p<.0001, |d|=.99$) was observed after the nature condition ($M=3, \textit{SD}=.74$) compared to the control condition ($M=2.43, \textit{SD}=.76$). Thus, H3 could be confirmed. However, as no significant differences ($t(26)=-.87, p=.2, |d|=.17$) could be observed for NA between nature ($M=1.17, \textit{SD}=.29$) and control ($M=1.2, \textit{SD}=.3$) conditions, H4 could not be confirmed.

### PSS

The total PSS score was tested for lower values after exposure to the nature condition ($M=19.33, \textit{SD}=6.53$) compared to the control condition ($M=21, \textit{SD}=6.29$). The result of the t-test ($t(26)=1.995, p=.03, |d|=.38$) supports our H5 hypothesis (see Fig. 7).

### IPQ

The IPQ total presence score was acquired to determine whether it was higher for the nature condition ($M = 3.82, \textit{SD}=.65$) compared to the control condition ($M = 2.65, \textit{SD}=.92$). As it can be seen in Fig. 8, the results support our H6 hypothesis ($t(26)=7.52, p<.0001, |d|=1.45$).

### SSQ

The total SSQ score was tested for lower values after exposure to the nature environment ($M=23.41, \textit{SD}=28.34$) compared to the control condition ($M=31.17, \textit{SD}=30.06$), which was supported by the results ($t(26)=-2.25, p=.02, |d|=.43$). Thus, H7 could be supported as well. Figure 9 shows the mean total scores for both environments.

## Discussion

In the present study, we examined the effects of exposure to an immersive computer-generated virtual nature environment and compared it to an immersive neutral control environment. The virtual nature consisted of a 3D model of a typical forest environment, whereas the control environment was an abstract replication of the virtual forest environment. In both environments, a virtual wooden cart was used to transport the users from the start to the end of the virtual road. The typical background noise of moving such a cart was integrated into both environments as well. In addition, the virtual nature environment included typical forest sounds in the background, whereas the control condition did not have such background sounds. As explained above in H1-H7, we hypothesized that exposure to virtual nature results in higher cognitive performance, higher perceived restorativeness, better mood, higher sense of presence, and lower stress and simulator sickness. To measure cognition, we employed assessed neuropsychological indicators, namely TMTA, TMTB, DSF and DSB.

We hypothesized that exposure to virtual nature would result in shorter completion times in TMT. The results of TMTB and its difference to TMTA support our hypothesis. This is consistent with the literature, in which performance in TMTB has shown improvements^86^. However, the non-significant difference in TMTA does not confirm our hypothesis. Nonetheless, this result is consistent with the previous literature, in which no significant improvement could be measured in the TMTA after exposure to nature^87,88^. As mentioned earlier, TMTA requires visuomotor abilities and processing speed whilst TMTB demands additional set-shifting skills. Thus, TMTB is cognitively more demanding which could be a reason for measurable effects after exposure to real or virtual natural environments. Likewise, it could be possible that the effect of nature on the TMTA is generally too small to be measured. Moreover, TMTB-A measures pure executive functioning by removing the visuomotor component. Thus, our results show that exposure to virtual nature is also able to improve pure higher-level cognitive functions assessed by TMTB-A.

Furthermore, our results showed significantly better performance in DSB after exposure to virtual computer-generated nature. However, no significant difference in performing DSF compared to the control condition could be observed. This is in contrast to the previous literature on nature exposure suggest improvements in both DSF and DSB^20,79,87–89^. Therefore, one might conclude that the benefits of exposure to computer-generated virtual nature may be measurable for more cognitively demanding tasks such as DSB. It is also worth noting that relatively more studies have employed DSB than DSF to measure benefits of nature on cognition^22^. Additionally, studies on DSF have compared exposure to nature with exposure to urban environments which have been reported to show a negative effect on cognitive performance^20,79,87,88,90–92^. In our study, however, we used an abstract neutral environment as the control condition which did not result in significantly different performance between nature and the control condition in DSF.

Overall, it can be stated that our participants were able to perform cognitively better after exposure to virtual nature compared to their performance after exposure to the control environment. They could concentrate more, were more attentive, and their working memory was superior after the virtual nature exposure than after experiencing our non-natural virtual environment. This could be associated with the perceived restorativeness (i.e., PRS) of the virtual nature environment which was significantly higher compared to the control environment. In addition to the total PRS score, four (Being Away, Fascination, Compatibility, and Coherence) out of its five sub-scales (German version of the PRS) were rated significantly higher for the virtual nature environment. For instance, participants found the virtual nature environment significantly more fascinating in which their attention could be drawn to many interesting things (Fascination sub-scale). Therefore, our findings validate the ART for exposure to natural environments in VR as well. Meaning that a short exposure of about 10 minutes to a computer-generated natural environment in VR is able to result in better cognition outside of VR compared to the same amount of exposure to a non-natural computer-generated virtual environment. However, this finding cannot be generalized to all human cognitive functions or long-term cognitive performance. Perceived restorativeness has been measured in previous studies using various instruments and questionnaires and yet, they all confirm restorativeness qualities of immersive computer-generated and $360^{\circ }$ videos of nature^50,51,56^. The only sub-scale of the measured perceived restorativeness scale in our study which was similarly rated for both environments was Scope (i.e., “That place is large enough to allow exploration in many directions”). This finding matches the passive navigation of the participants through the environment as they were not free to explore the environment on their own. Future studies could increase this aspect of perceived restorativeness by allowing active navigation using various techniques such as teleportation or redirected walking.

In accordance with the previous literature^59^, we also found improved positive affect as a result of exposure to virtual nature. However, we did not find a significant positive effect of virtual computer-generated nature on negative mood in comparison to the control environment. This is not completely in line with the previous literature as, for example, Newman et al.^59^ employed PANAS-X instrument and found significant reduction of negative affect after exposure to computer-generated nature environments in VR. Another example is a study by Mattila et al.^50^ who also employed the PANAS to measure affect and found significant increase of positive affect and significant decrease of negative affect. However, one has to consider that in these studies, ratings from before and after the exposure to the computer-generated virtual nature environments were compared, while in our study we assessed affect only after exposure to virtual nature and compared it to affect assessed after a control condition. Since the negative affect after exposure to any of these environments (nature and control) are very low (Control: $$ M=1.2 (SD=.3)$$, Nature: $$M=1.17 (SD=.29)$$), one could conclude that none of these environments induced a high level of negative affect in participants. Thus, our virtual control environment is not significantly worse than our virtual nature environment in terms of negative affect induction.

Perceived stress also showed significant reduction after exposure to virtual nature compared to the control condition. This finding validates the stress reduction theory (SRT, by Ulrich et al^16^). According to this theory, stress can be reduced by an encounter with unthreatening natural environments. As humans subconsciously prefer such environments, their sympathetic stress responses are reduced as a result of exposure. Our results showed that exposure to an immersive computer-generated virtual nature has similar effects and results in less perceived stress compared to a neutral control condition. This is inline with previous work on exposure to real-life $360^{\circ }$ videos of nature^36,52–54^.

Furthermore, we observed significantly higher levels of perceived sense of presence after the virtual computer-generated nature environment compared to the virtual control environment. Previous studies have assessed the sense of presence in the virtual nature environments with different questionnaires and in different experiment designs. For instance, Newman et al.^59^ observed a higher sense of presence in a more realistic virtual nature environment compared to a low realism virtual nature environment. In another study, a higher sense of presence in a virtual real-life $360^{\circ }$ video of nature was observed when it was compared to a virtual urban environment^37^. Also, Yeo et al.^27^ reported higher sense of presence for computer-generated virtual nature compared to $360^{\circ }$ videos.

Finally, our participants experienced significantly less symptoms of simulator sickness in their virtual nature exposure compared to the virtual control condition. It has to be mentioned that not all VR experiences involve induction of simulator sickness. In particular, when exposure to immersive VE is stationary and does not include any movements, observation of simulator sickness is rarely expected. For instance, most participants of a previous study did not experience any simulator sickness symptoms^59^ while most participants of another study which involved movements in the virtual nature environment reported high scores of simulator sickness^46^. In our study, participants were passively moved through the VE in a wooden cart. The total value of the simulator sickness questionnaire that was employed in our study can vary from 0 to 235.62. On this scale, the observed simulator sickness scores were M=31.17 for Control and M=23.41 for the Nature condition. One possible explanation could be that the camera movements in the virtual environments were quite smooth and did not have any sharp turns or accelerations. This resulted in reducing the overall amount of optic flow and sensory conflict known to contribute to motion sickness^93–96^. Nevertheless, these values were significantly less for the virtual nature compared to the control condition. However, we cannot rule out that the experienced simulator sickness may have had a confounding effect on the results. For instance, higher levels of simulator sickness may have been the reason for lower performance in cognitive tests or higher perceived stress after control condition.

Speaking of the limitations of this study, we should emphasize that the reported effects were measured after one time exposure. Therefore, no statement can be made about long-term effects of VR nature exposure. In order to make use of VR as an alternative tool for real nature exposure, future works should put this tool on trial and study its long-term effects.

Moreover, the virtual computer-generated nature in this study was limited to a forest environment. Thus, the observed effects cannot be generalized to other types of virtual nature settings such as mountains, underwater, ocean, etc. Neither can the effects be generalized to other environmental conditions such as weather, daylight, and background noise.

The VR experience in this study was also limited to the modalities of sight and sound. Future studies could enhance the immersion by including other senses such as smell and touch. Another limitation was that the users were not embodied in the VE. Granting a virtual body to users in future studies could enhance the sense of presence in the VE.

Furthermore, we did not undertake any screening with respect to general cognitive functioning or previous mental illness. The report on ADD/ADHD and eye disorders was based on single-item self-judgmental questions. These are further limitations of the current study. In the future, standard measures should be employed to ensure a homogeneous sample of participants. Moreover, with the student sample of our study with an average age of 28.15 years and no known history of mental disorders, the findings of this study cannot be generalized to other groups of users such as older adults or individuals with mental health conditions. Additionally, future studies may consider repeating this study in different cultural contexts, since our population was from a WEIRD (western, educated, industrialized, rich, and democratic) society.

A strength of this study is that it modeled virtual nature environment by means of a computer, which granted full control over the stimuli, in particular with respect to creating an abstract replication of our virtual forest. This is indeed an advantage of computer-generated virtual nature environments as opposed to $360^{\circ }$ videos. Once modeled, every element of the environment such as the time of the day, seasonal changes, weather condition, subtle movements, etc. can be manipulated and controlled.

Another strength of this study is to enable a comparison between a natural environment and a neutral environment with a comparable amount of objects in similar sizes and shapes which is not feasible in real-life experiments. The reason is that all real environments are either natural or man-made in urban environments. The results of this study extend our understanding of the key ingredients of a virtual natural environment which result in positive effects on cognitive functioning and affect. We could observe that it is not the space and the size and shape of the virtual objects, but rather other aspects unique to the depiction of natural elements that have to be investigated in further studies. The findings of this research could be of interest to a broad range of audiences including environmental psychologists as well as virtual environment designers (e.g., for interactive media such as games).

However, our efforts were directed towards providing an illusion of a familiar real nature environment to our participants from a northern German University. Therefore, our work is limited to what Keniger et al.^2^ call a Geographical Bias toward high latitudes in studies on the effects of interaction with nature. Thus, we are unable to provide any general recommendations about the naturality of virtual environments as we did not explore and compare all types of natural environments. This remains a topic for further research to provide evidence-based recommendations about the biodiversity of virtual natural environments and their key ingredients that elicit positive effects on cognitive functioning.

The outcome of this research has practical implications for VR-based interventions. We could show that the inclusion of 3D models of natural elements, such as trees, in a virtual environment creates an illusion that is able to elicit, among others, fascination and positive affect more than having the same amount of 3D models but non-natural in that environment. Thus, future VR experiences should consider careful designs of their virtual environments to benefit the most from the positive effects of such illusions.

All in all, this study provided solid evidence for the role of immersive virtual nature on improved mood and cognitive performance. Thus, individuals with limited access to real nature may benefit from its positive effects with the help of immersive VR. The induced illusion of being physically in virtual nature seems to elicit similar psychological mechanisms as being in real exposed to nature. The observed short-term effects should be the basis for future long-term studies in order to understand long-term effects of exposure to immersive virtual nature.

## Supplementary Information

Supplementary Information 1.

Supplementary Information 2.

## Data Availability

The datasets used and analysed during the current study will be available from the corresponding author on reasonable request.
