# Supplementary material for: Effects of exposure to immersive computer-generated virtual nature and control environments on affect and cognition
Source: Sci Rep. 2023 Jan 5;13:220. doi: 10.1038/s41598-022-26750-6 (PMC9815073; doi:10.1038/s41598-022-26750-6)
Supplement: Supplementary file 2 — Supplementary Information 2. [file 41598_2022_26750_MOESM2_ESM.pdf]

Supplementary Table of the paper entitled: Effects of Exposure to Immersive Computer-Generated Virtual Nature and Control Environments on Affect and Cognition

Fariba Mostajeran[1,*], Marvin Fischer[1], Frank Steinicke[1], and Simone Kühn[2,3]

[1]Universität Hamburg, Department of Informatics, Human-Computer Interaction Group, Hamburg, 22527, Germany
[2]University Medical Center Hamburg-Eppendorf, Clinic and Polyclinic for Psychiatry and Psychotherapy, Neural plasticity Group, Hamburg, 20246, Germany
[3]Max Planck Institute for Human Development, Lise Meitner Group for Environmental Neuroscience, Berlin, 14159, Germany
[*]fariba.mostajeran.gourtani@uni-hamburg.de

ABSTRACT

Previous research has shown that exposure to immersive virtual nature environments is able to induce positive affective and physiological effects. However, research on effects on cognitive performance is scarce. Additionally, the effects of virtual nature exposure compared to a virtual control environment with a comparable amount of virtual objects have not been examined so far. Therefore, we conducted an experiment with 27 participants to study the psychological effects of such an exposure. The virtual nature consisted of a 3D model of a typical forest environment, whereas the control environment was an abstract replication of the virtual forest environment. Both environments were compared with regard to their effects on cognitive performance, perceived restorativeness, mood, stress, sense of presence, and simulator sickness. The results showed that in comparison to the control environment, exposure to the virtual nature resulted in significantly higher cognitive performance, higher perceived restorativeness, higher positive affect, higher sense of presence, lower perceived stress, and lower simulator sickness.

Data analysis

According to the Shapiro-Wilk test, some of our data were normally distributed and some were not. Therefore, we decided to report the analysis based on parametric tests in order to not switch between statistical tests. For this reason, for each dependent variable and according to our hypothesis, a paired one-tailed t-test was calculated. The significance level was set at .05. Our analyses followed a preregistered protocol (`https://aspredicted.org/PNW_NYR`). Nevertheless, performing paired two-tailed tests on the measures did not change the significance of the results, except for PSS which yielded a p-value of .057, suggesting a trend toward our hypothesis (see Table 1). Additionally, Cohen's d was reported as the effect size for t-test which is commonly interpreted as small ($|d| = .2$), medium ($|d| = .5$), and large ($|d| = .8$) effects[1].

References

1. Cohen, J. A power primer. *Psychol. bulletin* **112**, 155 (1992).

	M (SD)		*One-tailed*		*Two-tailed*		
	Control	Nature	*t*	*p*	*t*	*p*	$\|d\|$
Trail Making Test (TMT)							
TMTA	23.49 (7.42)	22.89 (7.77)	-.5	.31	-.5	.62	.095
TMTB	50.13 (14.15)	45.57 (12.11)	-2.34	**.01**	-2.34	**.03**	.45
TMTB-A	26.64 (11.19)	22.68 (7.81)	-2.18	**.02**	-2.18	**.04**	.42
Digit Span Forward (DSF)	6.67 (1.49)	7 (1.24)	1.06	.15	1.06	.3	.2
Digit Span Backward (DSB)	6.3 (1.38)	6.85 (1.23)	2.96	**.003**	2.96	**.006**	.57
Perceived Restorativeness Scale (PRS)							
Total score	4.72 (1.11)	7.28 (1.16)	10.13	**<.0001**	10.13	**<.0001**	1.95
Being Away (BA)	4.87 (1.96)	7.94 (1.19)	10.27	**<.0001**	10.27	**<.0001**	1.98
Compatibility (COM)	2.81 (1.55)	7.19 (1.84)	10.26	**<.0001**	10.6	**<.0001**	2.04
Coherence (COH)	5.53 (1.72)	6.26 (1.19)	2.23	**.02**	2.23	**.03**	.43
Fascination (FA)	3.27 (1.83)	7.91 (1.58)	11.48	**<.0001**	11.48	**<.0001**	2.21
Scope (SCO)	7.11 (1.72)	7.11 (2.26)	0	.5	0	1	0
Positive and Negative Affect Schedule (PANAS)							
Positive Affect (PA)	2.43 (.76)	3 (.74)	5.16	**<.0001**	5.16	**<.0001**	.99
Negative Affect (NA)	1.2 (.3)	1.17 (.29)	-.87	.2	-.87	.39	.17
Perceived Stress Scale (PSS)	21 (6.29)	19.33 (6.53)	-1.99	**.03**	1.99	.057	.38
Igroup Presence Questionnaire (IPQ)	2.65 (.92)	3.82 (.65)	7.52	**<.0001**	7.52	**<.0001**	1.45
Simulator Sickness Questionnaire (SSQ)	31.17 (30.06)	23.41 (28.34)	-2.25	**.02**	-2.25	**.03**	.43

Table 1. *Mean (M)*, *Standard Deviation (SD)*, *t*- and *p*-values of one-tailed and two-tailed t-tests for all measurements. Significant p-values are represented in bold.
